# Portuguese Community Pharmacists' Attitudes to and Knowledge of Antibiotic Misuse: Questionnaire Development and Reliability

**DOI:** 10.1371/journal.pone.0090470

**Published:** 2014-03-05

**Authors:** Fátima Roque, Sara Soares, Luiza Breitenfeld, Cristian Gonzalez-Gonzalez, Adolfo Figueiras, Maria Teresa Herdeiro

**Affiliations:** 1 Health Sciences Research Centre, University of Beira Interior (CICS/UBI), Covilhã, Portugal; 2 Centre for Cell Biology, University of Aveiro (CBC/UA), Aveiro, Portugal; 3 Research Unit for Inland Development, Polytechnic Institute of Guarda (UDI/IPG), Guarda, Portugal; 4 Department of Preventive Medicine and Public Health, University of Santiago de Compostela, Santiago de compostela, Spain; 5 Consortium for Biomedical Research in Epidemiology & Public Health (CIBER en Epidemiología y Salud Pública - CIBERESP), University of Santiago de Compostela, Santiago de Compostela, Spain; 6 Centre for Health Technology & Information Systems Research (CINTESIS/FMUP), Porto, Portugal; 7 Health Technology Research Centre (CITS/CESPU), Gandra, Portugal; Cairo University, Egypt

## Abstract

**Objective:**

To develop and evaluate the reliability of a self-administered questionnaire designed to assess the attitudes and knowledge of community pharmacists in Portugal about microbial resistance and the antibiotic dispensing process.

**Methods:**

This study was divided into the following three stages: (1) design of the questionnaire, which included a literature review and a qualitative study with focus-group sessions; (2) assessment of face and content validity, using a panel of experts and a pre-test of community pharmacists; and, (3) pilot study and reliability analysis, which included a test-retest study covering fifty practising pharmacists based at community pharmacies in five districts situated in Northern Portugal. Questionnaire reproducibility was quantified using the intraclass correlation coefficient (ICC; 95% confidence interval) computed by means of one-way analysis of variance (ANOVA). Internal consistency was evaluated using Cronbach's alpha.

**Results:**

The correlation coefficients were fair to good (ICC>0.4) for all statements (scale-items) regarding knowledge of and attitudes to antibiotic resistance, and ranged from fair to good to excellent for statements about situations in which pharmacists acknowledged that antibiotics were sometimes dispensed without a medical prescription (ICC>0.8). Cronbach's alpha for this section was 0.716.

**Conclusions:**

The questionnaire designed in this study is valid and reliable in terms of content validity, face validity and reproducibility.

## Introduction

The emergence and spread of microbial resistance to antibiotics is an important public health problem and has been linked to increased and inappropriate use of antibiotics worldwide [1], [2]. This misuse of antibiotics, including self-medication, is known to contribute to infections with antibiotic-resistant micro-organisms, leading in turn to a rise in hospitalisations, length of hospital stays, mortality and health-care costs [3].

A review of antibiotic self-medication in Europe [4] repeatedly found high prevalences of self-medication in South and East European countries along with high levels of antibiotic resistance. Two major sources and practices involving self-medication were identified [4], namely: (1) over-the-counter dispensing of systemic antibiotics by community pharmacists; and, (2) the use of leftover antibiotics from previous treatments, resulting either from patients' non-compliance or from a larger number of tablets than needed being dispensed.

In Portugal, antibiotics may only be legally dispensed by community pharmacies under medical prescription. Although no studies could be located which specifically addressed the prevalence of self-medication with antibiotics and their acquisition from pharmacies without a medical prescription, a number of population-based studies show that this does indeed exist [5], [6].

It therefore follows that effective actions to improve antibiotic use and combat self-medication must necessarily include community pharmacists. This being so, it thus becomes essential to ascertain community pharmacists' knowledge of and attitudes to microbial resistances and antibiotic use, so that the pertinent educational interventions can be tailored to the task. No published, validated instrument designed for use on community pharmacists was however found. Accordingly, the aim of this study was to develop and assess the reliability of a self-administered questionnaire, purpose-designed to elicit the knowledge and attitudes of community pharmacists in Portugal about microbial resistance and the antibiotic dispensing process.

## Methods

### Ethics statement

Authorisation for this study (Permit No. 2886/2013) was obtained from the Portuguese Data Protection Authorities (*Comissão Nacional de Proteção de Dados/CNPD*). Written informed consent was obtained from all pharmacists who participated in the focus group study. As Portuguese community pharmacies are private entities, agreement to participate in the pilot study was obtained from the individual pharmacists, by sending them a cover letter explaining the study and asking them to complete and sign the accompanying questionnaire.

### Study population and sample selection

The study was conducted in a NUTS II (*Nomenclatura das Unidades Territoriais para Fins Estatísticos*/Nomenclature of Territorial Units for Statistics) area of Portugal defined by the Northern Regional Health Administration (*Administração Regional de Saúde do Norte, I.P./ARS-N*), and the target population included practising pharmacists based at community pharmacies in five Northern Portuguese districts (Braga, Bragança, Porto, Viana do Castelo and Vila Real).

### Questionnaire design

To obtain attitude scale-items, a qualitative study was conducted: this took the form of focus-group sessions held with community pharmacists to explore their perceptions, attitudes and knowledge about microbial resistance and antibiotic use [7]. The topic guide for this qualitative study with pharmacists was based on a review of the literature [7].

### Face and content validity

Two clinical psychology experts and one Portuguese language expert evaluated face-validity parameters, such as the grammar, syntax, organisation, appropriateness and logical sequence of the statements [8].

Content validity was assessed by an expert panel consisting of three pharmacologists and three specialist pharmacists (the latter being a grade awarded by the Portuguese Pharmaceutical College). This appraisal stage [9] is fundamental for assessing the accuracy, clinical terminology, completeness and meaning of items.

To clarify possible problems of comprehension with any questionnaire item, a pre-test was conducted on ten community pharmacists, who were invited to complete the questionnaire and comment on any difficulties experienced in interpreting the respective items.

### Pilot study and reliability analysis

A test-retest study was conducted using a convenience sample of 50 community pharmacists drawn from all districts in Portugal's Northern Region. Questionnaires were delivered together with a cover letter, outlining the study objectives and highlighting the importance of each pharmacist's participation.

To assess reliability [10], questionnaires were delivered to each pharmacist twice, at an interval of two to four weeks, in line with the scientific literature [11] and previous studies [12].

#### Statistical analysis

Questionnaire reproducibility (degree of agreement among answers) was quantified using the intraclass correlation coefficient (ICC; 95% confidence interval) [13], [14] computed by means of one-way analysis of variance (ANOVA) [10].

The internal consistency of the group of questions on the dispensing of unprescribed antibiotics by pharmacists, was evaluated using Cronbach's alpha [15], [16].

## Results

**Figure 1:**
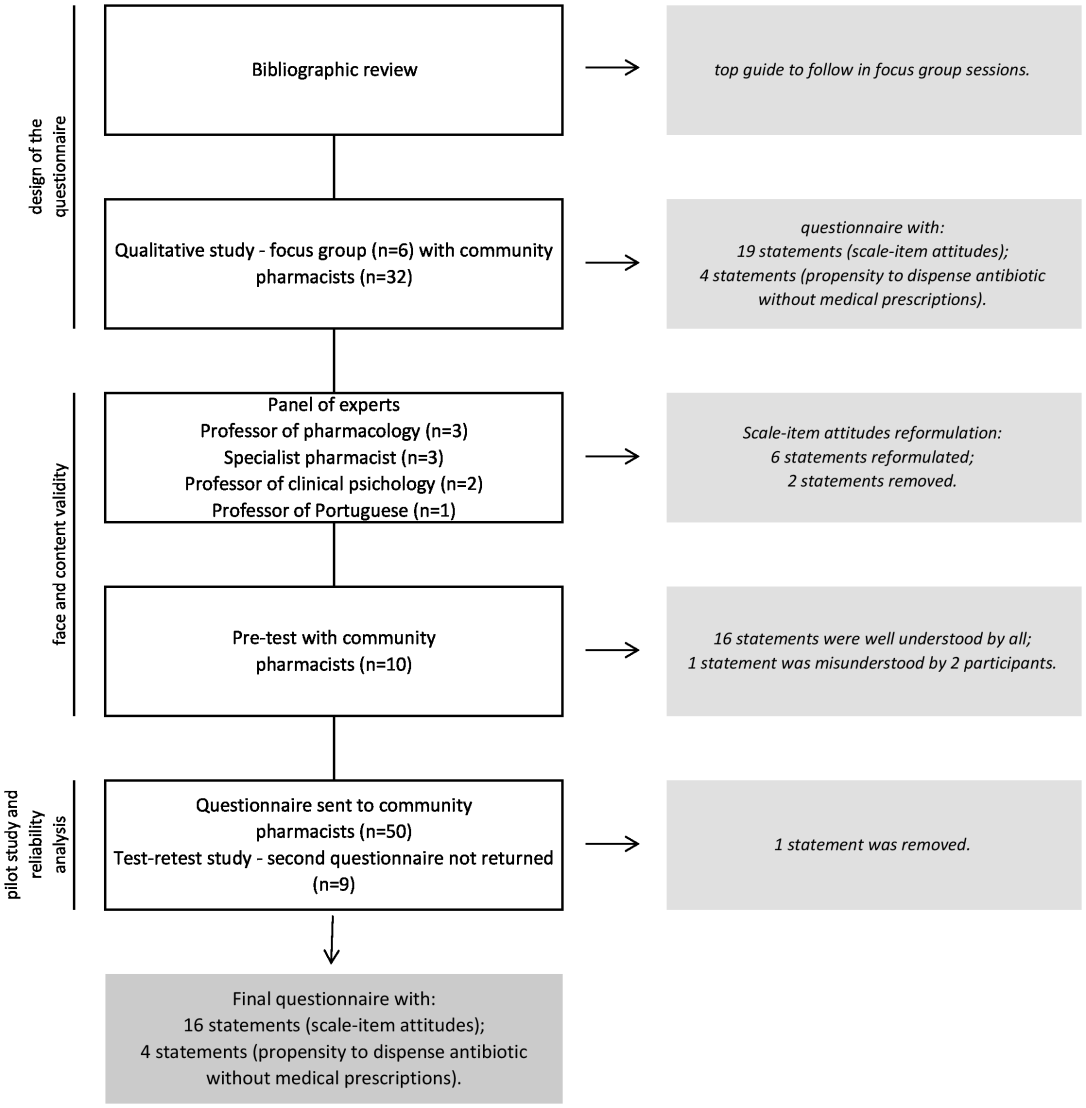
Flow Chart.

The different stages of the study and the outcomes obtained at each stage are shown in Figure 1.

### Questionnaire design

The six focus-group sessions that were held during the qualitative study and involved a total of 32 pharmacists, enabled us to explore pharmacists' perceptions, knowledge and attitudes about microbial resistances and antibiotic use [7].

Data collected from this qualitative study were used to draw up a structured questionnaire containing scale-items that reflected pharmacists' attitudes to and knowledge of microbial resistance, antibiotic use and the antibiotic dispensing process.

The questionnaire obtained at this stage was one sheet long (two pages), divided into the following five sections: Section 1, containing a box with instructions on how to complete the form; Section 2, containing nineteen statements (scale-items) regarding pharmacists' attitudes to and knowledge of microbial resistance and antibiotic use, followed by a horizontal, continuous visual analogue scale (VAS) for respondents to mark with a cross; Section 3, containing four statements about the process of dispensing antibiotics without a medical prescription, followed by a VAS for respondents to mark with a cross; Section 4, containing personal and professional data, such as age, gender, workplace, job function and mean number of antibiotics dispensed; and, Section 5, containing a request to professionals to make suggestions about antibiotic use and microbial resistance.

### Face and content validity

Clinical psychology and Portuguese language experts assessed the grammar, syntax, organisation, appropriateness and logical sequence of scale-items. Initially, Section 2 of the questionnaire contained 19 statements but after evaluation by the experts, 6 statements were redrafted and 2 statements were deleted. The statements deleted were, “Antibiotics are over prescribed” and “In situations where the patient is known, antibiotics may sometimes be sold without a medical prescription”, because the experts felt that these two items were already included in other statements and in Section 3.

Expert pharmacologists and specialist pharmacists evaluated the accuracy, clinical terminology, completeness and meaning of all questions, and considered that the questionnaire was well constructed and included important issues, such as therapeutic compliance and drug-drug interactions that could influence the development of resistance.

The statement S11, “When antibiotics are returned to Valormed, patients should be alerted to the consequences of not complying with the treatment”, gave rise to several problems: the clinical psychology expert did not understand the qualitative meaning of the statement, and of the ten pharmacists who completed the questionnaires designed to assess respondents' comprehension of each statement, two commented that the content of a Valormed bag cannot be evaluated (seen or commented upon) by pharmacists at the time it is delivered (Valormed is a Portuguese system which is available at Portuguese pharmacies for collecting medicinal packaging and unused or expired medicines).

Even so, it was decided that, since the statement sought to respond to a major concern raised by those participating in the focus-group sessions, it would not be deleted but would instead be maintained during the pilot study.

### Pilot study and reliability analysis

Of the 50 pharmacists initially invited, 41 completed the study (82%), and 9 (18%) did not complete the questionnaire used for retest purposes.

#### Reproducibility

ICCs were determined for all the statements contained in Sections 2 and 3 of the questionnaire (Tables 1 and 2).

**Table 1:** Intraclass correlation coefficients (ICCs) assessed for pharmacists' attitudes (Section 2 of the questionnaire).

Statements about knowledge of and attitudes to microbial resistance to antibiotics	ICC (95% CI)	*p*-value
**S1: Antibiotic resistance is an important Public Health problem of ours.**	0.778 (0.586–0.881)	<0.001
**S2: The fact of a patient taking an antibiotic increases the risk of developing resistance.**	0.777 (0.576–0.882)	<0.001
**S3: In all cases where antibiotics are dispensed, it is essential that patients be advised about complying with the treatment.**	0.678 (0.389–0.830)	<0.001
**S4: An important cause of appearance of antibiotic resistance is long-term prescription of new molecular entities.**	0.789 (0.606–0.887)	<0.001
**S5: When dispensing, possible interactions between the antibiotic and other drugs that the patient is taking should be assessed.**	0.463 (−0.002–0.713)	0.025
**S6: Antibiotics are sometimes dispensed without medical prescription because the patient is known to have difficulty in obtaining a medical consultation.**	0.760 (0.552–0.872)	<0.001
**S7: Two of the main causes of the appearance of antibiotic resistance are patient self-medication and antibiotic misuse.**	0.586 (0.228–0.779)	0.003
**S8: Antibiotics are sometimes dispensed, even when it is known that they are not indicated, because there is no time to explain the reason why they are not called for.**	0.469 (0.009–0.716)	0.023
**S9: If a patient feels that he/she needs antibiotics and these are not dispensed, he/she will easily obtain the prescription and could accuse us of having delayed the treatment.**	0.635 (0.319–0.805)	0.001
**S10: I am convinced that new antibiotics will be developed to solve the problem of resistance.**	0.608 (0.269–0.791)	0.002
* **S11: When antibiotics are returned to Valormed** a **, patients should be alerted to the consequences of not complying with the treatment.**	0.677 (0.392–0.829)	<0.001
**S12: The use of antibiotics in animals for human consumption is an important cause of appearance of new resistance to pathogenic agents in humans.**	0.610 (0.272–0.791)	0.002
**S13: Antibiotics are sometimes prescribed without medical prescription because the patient is known to have neither the time nor the money to see a physician.**	0.794 (0.615–0.810)	<0.001
**S14: If a patient feels that he/she needs antibiotics and these are not dispensed, he/she will easily manage to obtain them at another pharmacy.**	0.732 (0.501–0.857)	<0.001
**S15: Antibiotic prescribing should be more closely controlled.**	0.439 (−0.056–0.702)	0.0036
**S16: Dispensing antibiotics without prescription should be more closely controlled.**	0.796 (0.614–0.893)	<0.001
**S17: The phenomenon of resistance to antibiotics is mainly a problem in hospital settings.**	0.541 (0.143–0.754)	0.007

a VALORMED is a system for collecting medicinal packaging and unused or expired medicines.

*This item was removed from the final questionnaire.

**Table 2:** Intraclass correlation coefficients (ICCs) assessed for situations in which pharmacists acknowledge that antibiotics are sometimes dispensed without a medical prescription (Section 3 of the questionnaire).

IN CASES WHERE THE PATIENT IS KNOWN, ANTIBIOTICS ARE SOMETIMES DISPENSED WITHOUT A MEDICAL PRESCRIPTION IN THE FOLLOWING SITUATIONS:	ICC	*p*-value	Cronbach's alpha, if item deleted
**1. Dental diseases and ailments (e.g., dental abscesses).**	0.823 (0.663–0.907)	<0.001	0.682
**2. Upper respiratory infections (e.g., otitis media, pharyngitis, etc.).**	0.454 (−0.035–0.713)	0.032	0.597
**3. Urinary infections (cystitis).**	0.860 (0.732–0.927)	<0.001	0.507
**4. Any infection in which the patient undertakes to bring the prescription.**	0.733 (0.488–0.861)	<0.001	0.773

The correlation coefficients for statements in Section 2 (Table 1) exceeded 0.4 (*p*<0.05) for all attitudes, and ranged from 0.439 (*p*<0.01) (statement S15: “Antibiotic prescribing should be more closely controlled”) to 0.796 (*p*<0.001) (statement S16: “Dispensing antibiotics without prescription should be more closely controlled”).

The correlation coefficients for statements in Section 3 relating to antibiotic dispensing without a prescription (Table 2) exceeded 0.4 (*p*<0.05), and ranged from 0.454 (*p*<0.05) to 0.860 (*p*<0.001).

#### Internal consistency

Cronbach's alpha for Section 3 of the questionnaire was 0.716. To obtain one scale with higher internal reliability, statements were deleted one at a time until a group of items with the highest Cronbach's alpha values was obtained (Table 2).

#### Suggestions in Section 5 of the questionnaire

Six of the pharmacists included in this study added comments under the “suggestions” item, as requested by the questionnaire: in three cases these involved suggestions for new topics, such as diagnostic tests on susceptibility to antibiotics and the availability of information on the interaction between antibiotics and other drugs; the remainder related to the wording of statement S11.

## Discussion

This is the first study to design and validate an instrument for measuring pharmacists' attitudes to antibiotic misuse. Our questionnaire showed itself to be a valid, reliable and reproducible instrument for measuring pharmacists' attitudes to and knowledge of microbial resistance and antibiotic dispensing behaviour.

Attitude and knowledge scale-items were measured using an 8-centimetre long, unnumbered VAS, with answers scored from total disagreement to total agreement. Visual analogue scales are derived from the Likert scale, have shown themselves to be more sensitive for detecting small differences, and might possibly be more reliable and valid [17], [18]. VAS-based questionnaires have been used in other studies to assess pharmacists' attitudes to adverse drug reaction (ADR) reporting [19], with the attitudes identified then being used to develop highly successful interventions to improve ADR reporting [20].

The results show that our questionnaire was well accepted and understood by pharmacists and that it enabled reliable results to be obtained. Furthermore, the test-retest study indicates that the responses to both Section two and Section three of the questionnaire displayed an acceptable degree of reproducibility. According to the values cited by Rosner [14], the ICCs obtained in our study show fair to good (ICC>0.4) or excellent (ICC>0.7) reproducibility for all scale-items. Despite the fact that Statement 11 displayed fair to good reproducibility (ICC 0.677; *p*<0.001), it was nevertheless removed for further analysis because comments made during the test-retest study reinforced the opinion voiced by the clinical psychologist and pharmacists during the face- and content-validity stage. Statement 11 was intended to evaluate pharmacists' concern about prescription compliance but we concluded that this item was liable to misinterpretation. We therefore decided to eliminate it and propose a final questionnaire, containing 16 attitude and knowledge scale-items.

The statements in Section 3 (situations in which pharmacists acknowledged that antibiotics were sometimes dispensed without a medical prescription) showed a satisfactory level of internal consistency [15], thereby indicating that all items (n = 4) measured the same concept [16], i.e., the propensity to dispense antibiotics without a medical prescription. Cronbach's alpha was not calculated for attitude and knowledge scale-items in Section 2 of the questionnaire because this scale was designed to assess different attributes and to apply a measure of internal consistency would thus not make sense [16].

Our study's high degree of reliability may be due to data obtained from a previous qualitative study in the form of focus-group sessions with community pharmacists, and/or to one or more of the following: (1) the objectivity of the questions being first assessed by experts in linguistics and psychology; (2) the interest shared by the pharmacists in antibiotic resistance issues; (3) the fact that the questionnaire was also assessed by an expert panel of pharmacologists and specialist pharmacists; (4) the use of a continuous VAS; and, (5) the 2- to 4-week interval between two responses on the same subject [12].

The major limitations of this study lie in the fact that the pilot-study sample was not only very small but was a convenience sample, meaning in turn that the attitudes identified could not be extrapolated to a larger population. However, the main goal of the pilot study was to assess the reliability of the questionnaire developed during stages 1 and 2 of the study, and for this purpose, the most widely used approach relies on sample sizes of n≥30 and sample selection by a convenience method [21], [22].

Some studies focus on self-medication as a factor involved in the development of antibiotic resistance [4], while other authors point to the sale of antibiotics without a prescription as a reality in Europe [3], [23] and pharmacies therefore being another link in the chain of antibiotic resistance [24]. Yet, we located only one study, conducted on community pharmacists in Southern Thailand [25], which reported that attitudes to microbial resistance could influence the dispensing of antibiotics for upper respiratory infections. There are no other studies in the literature addressing the validation of scales designed to assess pharmacists' attitudes vis-à-vis this important issue, thus ruling out comparisons between our study and others.

In a review [8] of the nature, validity, and reliability of measurement scales designed to assess factors linked to antibiotic misuse/overuse, the authors included 27 studies with scales aimed at patients/parents, 13 studies with scales aimed at physicians, and 15 studies with scales aimed at physicians and patients/parents: scales aimed at pharmacists were not included, revealing the lack of studies targeted at identifying community pharmacists' attitudes to and knowledge of antimicrobial resistance and the antibiotic dispensing process.

Despite the lack of studies into community pharmacists' attitudes to microbial resistance and antibiotic use, some studies, undertaken in hospital settings to compare the attitudes of pharmacists and physicians to hospital antibiotic policies [26], stress the important role played by pharmacists in hospital-guideline implementation [27], [28] and the therapeutic decision-making process, even in primary care [29].

As mentioned above, pharmacists have an important role to play in policy and guideline implementation and patient management of medication, i.e., antibiotic use, but there are no validated tools for measuring their attitudes to these issues.

Accordingly, we feel that the instrument developed and validated in this study, in the form of a self-administered questionnaire, could prove very useful in future studies undertaken for the purpose of ascertaining community pharmacists' attitudes to and knowledge of microbial resistance and antibiotic use. Educational interventions addressing these attitudes could be designed to improve antibiotic dispensing practices, with the aim of combating microbial resistance and promoting public health.
