# Correction: Portuguese Community Pharmacists' Attitudes to and Knowledge of Antibiotic Misuse: Questionnaire Development and Reliability

**DOI:** 10.1371/journal.pone.0145973

**Published:** 2015-12-23

**Authors:** Fátima Roque, Sara Soares, Luiza Breitenfeld, Cristian Gonzalez-Gonzalez, Adolfo Figueiras, Maria Teresa Herdeiro

The following information is missing from the Funding section: This study was co-financed by Fundo Europeu de Desenvolvimento Regional (FEDER) through the Programa Operacional Factores de Competitividade (COMPETE) Program.
